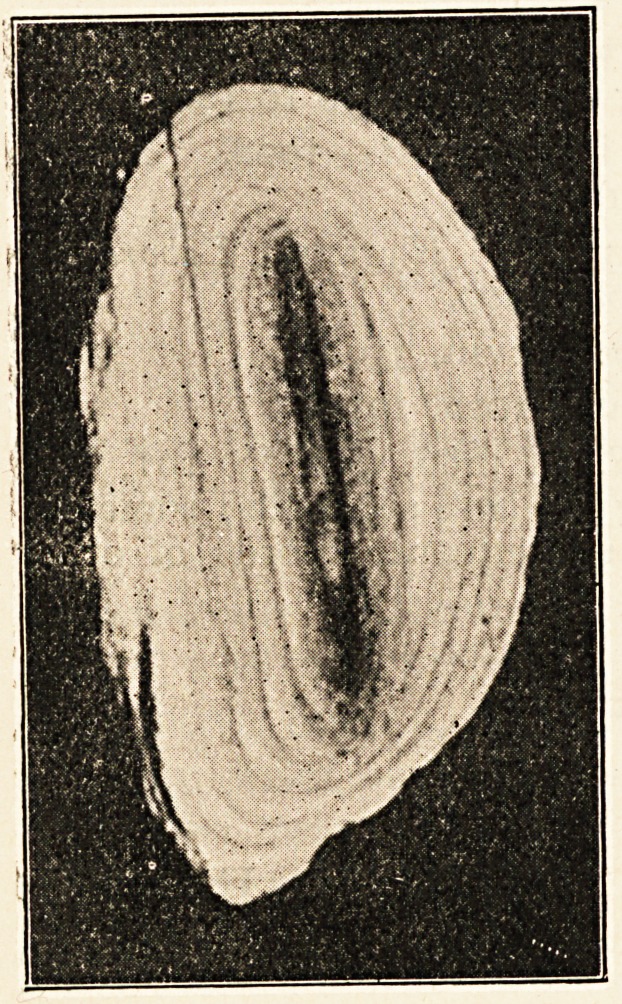# A Case in Which a Stone Formed in the Urethra around a Piece of Wood Introduced into the Urethra Twenty-Six Years before the Removal of the Stone
1Specimen shown at a meeting of the Bristol Medico-Chirurgical Society, March 9th, 1910.


**Published:** 1910-06

**Authors:** Charles A. Morton

**Affiliations:** Professor of Surgery in the University of Bristol, and Senior Surgeon to the General and Children's Hospitals.


### A CASE IN WHICH A STONE FORMED IN THE URETHRA AROUND A PIECE OF WOOD INTRODUCED INTO THE URETHRA TWENTY-SIX YEARS BEFORE THE REMOVAL OF THE STONE.[1]

BY

CHARLES A. MORTON, F.R.C.S.,

*Professor of Surgery in the University of Bristol, and Senior Surgeon to the General and Children's Hospitals.*

---

IN July, 1909, I was asked by Dr. A. Peake to see a man, aged 48, who had a sinus in the perineum. When a probe was passed along this it struck a stone in the urethra. The history of the case was a very remarkable one. Twenty-six years before we saw

[1] Specimen shown at a meeting of the Bristol Medico-Chirurgical Society, March 9th, 1910.

him he had retention of urine, and in order to relieve himself he passed a kind of bougie which he made of wood. The end of this broke off in his urethra, but curiously enough it did relieve the retention. The broken-off end of the wooden bougie, however, remained in the urethra. He had had difficulty with micturition at times since, and had kept in the house a No. 4 metal catheter, which he passed when the difficulty was marked. In doing so, he told us, he struck the piece of wood. He really struck the stone, but knowing nothing about stones in the urethra, he imagined it to be the piece of wood. A few weeks before we saw him a sinus formed in the perineum, and Dr. Peake had opened another abscess there a few days before we saw him together. There was a large mass of induration in the perineum, more to the right than the left. In this was the earlier sinus and Dr. Peake's opening. Pus and urine flowed from both. A No. 3 silver catheter passed into the bladder, but with considerable difficulty. It grated over the stone. The urine was purulent but not offensive.

A few days later I removed the stone. The mass of induration was very largely due to the size of the stone. It lay in a pouched urethra, distinctly more to one side of the middle line than the other. On cutting open the stone a piece of wood was discovered as its nucleus. This seems to have been prepared by nature for photographic purposes. Some constituent of the urine had stained its outer layer red, and this has, of course, come out as a black line in the photograph, as if the piece of wood had been drawn in with ink. The photograph represents the stone one-eighth of an inch larger than it is. My efforts to get it exactly the actual size were unsuccessful.

## Figures and Tables

**Figure f1:**